# Pharmacologic Treatments and Supportive Care for Middle East Respiratory Syndrome

**DOI:** 10.3201/eid2606.200037

**Published:** 2020-06

**Authors:** Taylor Kain, Patrick J. Lindsay, Neill K.J. Adhikari, Yaseen M. Arabi, Maria D. Van Kerkhove, Robert A. Fowler

**Affiliations:** University of Toronto, Toronto, Ontario, Canada (T. Kain, P.J. Lindsay, N.K.J. Adhikari, R.A. Fowler);; Harvard University, Boston, Massachusetts, USA (P.J. Lindsay);; Sunnybrook Health Sciences Center, Toronto (N.K.J. Adhikari, R.A. Fowler);; King Saud Bin Abdulaziz University for Health Center, Riyadh, Saudi Arabia (Y.M. Arabi);; King Abdullah International Medical Research Center, Riyadh (Y.M. Arabi);; World Health Organization, Geneva, Switzerland (M.D. Van Kerkhove)

**Keywords:** Middle East respiratory syndrome, MERS, coronavirus, CoV, coronavirus infections, acute respiratory distress syndrome, severe acute respiratory syndrome, systematic review, antivirals, supportive care, interferon, ribavirin, corticosteroids, convalescent plasma, intravenous immunoglobulin, respiratory infections, viruses, zoonoses

## Abstract

Available animal and cell line models have suggested that specific therapeutics might be effective in treating Middle East respiratory syndrome (MERS). We conducted a systematic review of evidence for treatment with pharmacologic and supportive therapies. We developed a protocol and searched 5 databases for studies describing treatment of MERS and deaths in MERS patients. Risk of bias (RoB) was assessed by using ROBINS-I tool. We retrieved 3,660 unique citations; 20 observational studies met eligibility, and we studied 13 therapies. Most studies were at serious or critical RoB; no studies were at low RoB. One study, at moderate RoB, showed reduced mortality rates in severe MERS patients with extracorporeal membrane oxygenation; no other studies showed a significant lifesaving benefit to any treatment. The existing literature on treatments for MERS is observational and at moderate to critical RoB. Clinical trials are needed to guide treatment decisions.

Middle East respiratory syndrome (MERS), which is now known to be caused by MERS coronavirus (MERS-CoV), was first reported in September 2012 in Saudi Arabia (*1*). Since then, it has spread to 26 other countries (*2*). As of November 30, 2019, a total of 2,494 confirmed cases and 858 deaths had been reported to the World Health Organization (WHO); the case-fatality rate was 34.4% (*3*). To date, all cases have been linked to travel or residence in the Arabian Peninsula. MERS-CoV is a human betacoronavirus that is found in humans and dromedary camels and is similar to other human coronaviruses (e.g., severe acute respiratory syndrome coronavirus [SARS-CoV] and SARS-CoV-2, the cause of coronavirus disease [COVID-19]) (*4*). Infected patients generally have fever, cough, dyspnea, and abnormal chest imaging (*5*). Many patients have onset of respiratory failure and require noninvasive ventilation (NIV) or invasive mechanical ventilation; advanced supportive care techniques, such as extracorporeal membrane oxygenation (ECMO), have been used. Most of these patients are cared for in an intensive care unit (ICU).

No vaccination against MERS-CoV infection exists, and WHO and the US Centers for Disease Control and Prevention (CDC) recommend general infection prevention measures when caring for patients (*1*,*6*). As with other coronaviruses, no evidence-based recommended pharmacologic therapy for the treatment of MERS-CoV infection exists; however, limited data from available animal and cell line models have led to multiple different combinations of antiviral drugs and other adjunctive therapies to be proposed and used in humans (*7*,*8*). We conducted a systematic review to summarize the current evidence base for treatment of MERS, including specific treatments against MERS, adjunctive pharmacologic therapies, and supportive care.

## Methods

### Literature Search and Selection Criteria

We developed a protocol that considered the Preferred Reporting Items for Systematic Reviews and Meta-Analyses checklist (*9*), which was registered with the International Prospective Register of Systematic Reviews (reference no. CRD42018114622). We searched for relevant studies in 5 databases (MEDLINE, PubMed, Embase, Cochrane, and Cumulative Index to Nursing and Allied Health Literature) in August 2018 and updated the results in October 2019 (Appendix Figure). We imposed no language restrictions. We also searched reference lists of studies included in the review, as well as ClinicalTrials.gov for any ongoing or completed trials.

We imported all abstracts into Covidence (Veritas Health Innovation, https://www.covidence.org) for review. After we removed duplicates, 2 authors (T.K. and P.L.) independently and in duplicate screened titles and abstracts of references generated from the literature search. The population studied was patients of any age admitted to a hospital with laboratory-confirmed MERS. We included studies with >5 cases in patients who received a therapy targeting MERS or that examined supportive care for MERS. Specific and supportive care therapies included, but were not limited to, antiviral drugs, immunomodulatory medications (e.g., corticosteroids), antibody-based pharmaceuticals, and alternative oxygen-delivery therapies (e.g., ECMO and NIV). We included studies that reported our primary outcome of interest, death at any point of illness. We also recorded information regarding secondary outcomes where available, including hospital length of stay, ICU length of stay, mechanical ventilation days, and adverse events. Eligible studies included randomized controlled trials (RCTs), nonrandomized single-arm intervention studies (with or without a control group), prospective and retrospective cohort studies, and case series. We used the term “cohort studies” to describe those in which an association between an exposure and outcome was reported for an eligibility criteria–defined complete group of consecutive patients, and only if the exposure was relevant to this review. If exposure was not relevant, then we reclassified the study as a case series. We intended to only include studies with a comparator or control group, but because of the varying quality of papers retrieved, we deviated from our original methodologic plan and included any study describing patients given a treatment of interest, even if no specific control group was available. We excluded all preclinical studies (i.e., those performed on animals or human cell lines).

Two review authors (T.K. and P.L.) compared screening results and discussed differences. Any disagreement on eligibility was resolved through consensus with 2 other authors (R.A.F., N.K.J.A.). We constructed a PRISMA diagram of the included studies (Figure).

**Figure:**
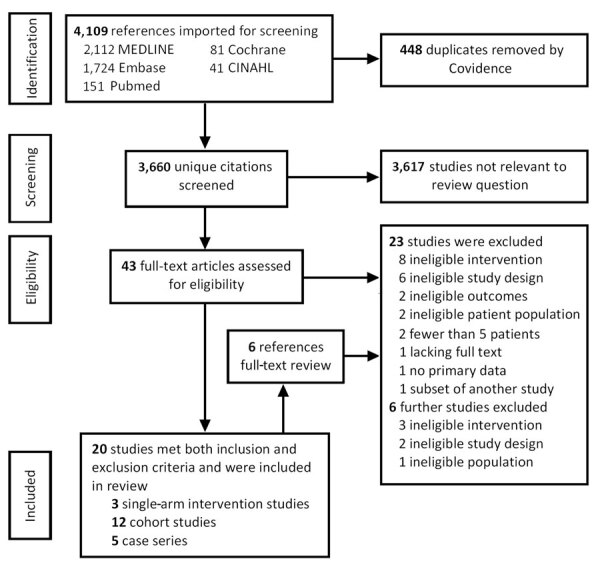
Preferred Reporting Items for Systematic Reviews and Meta-Analyses diagram of literature search results, screening performed, and reasons for exclusion of full text reviews from a systematic review of evidence for MERS treatment with pharmacologic and supportive therapies. CINAHL, Cumulative Index to Nursing and Allied Health Literature.

### Data Extraction

For each included study, 2 authors (T.K. and P.L.) independently and in duplicate extracted data, including publication year, location of study, patient location (e.g., ICU or ward), dates of subject enrollment, study design, baseline characteristics (e.g., age and underlying conditions), study interventions and co-interventions, and clinical outcomes of interest (including death). Given the small pool of patients, we observed a substantial overlap in patients reported among the studies; therefore, we estimated the number of unique patients among all studies, attempting to contact primary authors for clarification when needed.

### Quality Assessment and Statistical Analysis

Two authors (T.K. and P.L.) assessed the risk of bias (RoB) by using the ROBINS-I tool for nonrandomized intervention and cohort studies (*10*). Other authors (R.A.F. and N.K.J.A.) verified selected methodologic details of these studies. We did not assess the methodologic quality of case series in the absence of a validated tool. We assessed the overall certainty of evidence by using the GRADE framework (*11*), considering RoB, inconsistency, imprecision, and publication bias of trials.

## Results

### Search Results

We retrieved 4,108 citations, of which 448 were duplicates, leaving 3,660 unique citations; we determined that 3,167 were not relevant. After full-text screening the remaining 43 studies, we found 20 that met eligibility criteria (Figure): 3 nonrandomized single-arm intervention studies (*12*–*14*), 12 prospective and retrospective cohort studies (*15*–*26*), and 5 case series with >5 patients (*27*–*31*). We then reviewed the references of these papers; an additional 6 papers underwent full review, but none met eligibility. No RCTs had been completed at the time of review.

The included studies enrolled patients during September 2012–March 2018. We estimated the number of unique patients among studies to be 678–865, representing 31%–40% of the 2,189 patients with MERS during that period (*32*). The diversity of different specific therapies studied and the substantial overlap in patients among studies precluded formal meta-analyses.

### Patient and Study Characteristics

Of the 20 included studies, 19 were conducted in Saudi Arabia; 1 was conducted in South Korea during the 2015 outbreak (Table 1). During the trials, the median or mean age of patients was 45–66 years (Table 2). Many had >1 underlying condition; diabetes and chronic kidney disease were the most common. Mortality rates in studies were high, ranging from 20% to 100%. An overall mortality rate could not be accurately calculated because of the substantial overlap in patients included in multiple studies.

**Table 1:** Demographic information and main intervention (where applicable) for studies included in a systematic review of evidence for MERS treatment with pharmacologic and supportive therapies, by type of study*

Reference	Location (no. centers)	Study period	No. patients	Intervention group	Comparator group	Primary outcome
Nonrandomized, single-arm intervention study with historical comparisons						
(*12*)	Jeddah, Saudi Arabia (1)	April–June 2014	32	IFN-β1a (May–Jun); 11 patients	IFN-α2a (Apr); 13 patients	Mortality rate (unspecified)
(*13*)	Saudi Arabia (5)	Sept 2012–Dec 2015	35	ECMO; 17 patients	No ECMO 18 patients	90-d mortality rate
(*14*)	Riyadh, Saudi Arabia (1)	Oct 2012–May 2014	70 (44 included)	RBV/IFN-α2a; 20 patients	Supportive care 24 patients	14-d and 28-d mortality rate
Prospective cohort study						
(*15*)	Seoul, South Korea (3)	May–July 2015	30	NA	NA	NA
(*16*)	Jedda, Saudi Arabia (1)	Mar–Jun 2014	8	NA	NA	NA
Retrospective cohort study						
(*17*)	Saudi Arabia (14)	Sept 2012–Oct 2015	309	Steroids; 151 patients	No steroids 157 patients	90-d all-cause mortality rate
(*18*)	Saudi Arabia (14)	Sept 2012–Oct 2015	330 MERS	MERS	Non-MERS SARI	90-d mortality rate
(*19*)	Riyadh, Saudi Arabia (1)	Oct 2012–May 2014	70 (31 included)	NA	NA	NA
(*20*)	Al-Madinah City, Saudi Arabia (2)	Mar–May 2014	29	NA	NA	NA
(*21*)	Jeddah, Saudi Arabia (1)	April–May 2014	14	NA	NA	Survival at 1 y
(*22*)	Jedda, Saudi Arabia (1)	Jan–Dec 2014	51	NA	NA	NA
(*23*)	Riyadh, Saudi Arabia (1)	April 2014–Mar 2018	314	NA	NA	Mortality rate (unspecified)
(*24*)	Saudi Arabia (14)	Sept 2012–Jan 2018	349	Macrolides	No macrolides	90-d mortality rate
(*25*)	Saudi Arabia (14)	Sept 2012–Oct 2015	302	NIV	Invasive ventilation	90-d mortality rate
(*26*)	Saudi Arabia (14)	Sept 2012–Jan 2018	349	RBVIFN	No RBV/IFN	90-d mortality rate
Case series without evaluation of treatments						
(*27*)	Al-Hasa, Saudi Arabia (1)	April–May 2013	5	NA	NA	NA
(*28*)	Riyadh, Saudi Arabia (1)	Dec 2012–Aug 2013	11	NA	NA	NA
(*29*)	Al-Hasa, Saudi Arabia (1)	April 2012–Nov 2016	107	NA	NA	NA
(*30*)	Riyadh, Saudi Arabia (1)	Before Oct 2014	6	NA	NA	NA
(*31*)	Riyadh, Saudi Arabia (1)	July–Oct 2015	63 (8 included)	NA	NA	NA

*ECMO, extracorporeal membrane oxygenation; IFN, interferon; MERS, Middle East respiratory syndrome; NA, not applicable; NIV, noninvasive ventilation; NS, nonsurvivors; RBV, ribavirin; SARI, severe acute respiratory infection.

**Table 2:** Underlying conditions, age of study populations, overall mortality rates, and mortality rates by intervention (where applicable) for studies included in a systematic review of evidence for MERS treatment with pharmacologic and supportive therapies, by type of study*

Reference	Age, y	Underlying conditions			Intervention			
		≥1	Diabetes mellitus	CKD		Mortality rate		
						Intervention	Comparison	Total
Nonrandomized, single-arm intervention study with historical comparisons								
(*12*)	66 (median)	NR	15 (47%)	16 (50%) 6 (19%) on dialysis	IFN-β1a vs IFN-α2a	64% IFN-β1a	85% IFN-α2a	69%
(*13*)	46 (median ECMO); 50 (median no ECMO)	NR	18 (51%)	5 (14%)	ECMO	65%	100%	83%
(*14*)	66 y (mean)	NR	30 (68%)	11 (26%)	RBV + IFN-α2a	14d: 30%; 28d: 70%	14d: 71%,28d: 83%	52% at 14 d; 77% at 28 d
Prospective cohort study								
(*15*)	49 (mean)	11 (47%)	4 (13%) NS 1 (25%)	1 (3%) NS 0 (0%)	NA	NA	NA	20%
(*16*)	57 (median)	NR	5 (63%)	NR	NA	NA	NA	75%
Retrospective cohort study								
(*17*)	58 (mean steroids); 55 (mean no steroids)	132 (87%) steroids 115 (73%) no steroids	87 (58%) steroids 69 (44%) no steroids	43 (29%) steroids 47 (30%) no steroids	Steroids	90-d 74% Hospital 78%	58% 90-d Hospital 58%	66%
(*18*)	58 (median)	265 (80%) NS 199 (75%)	162 (49%) NS 124 (77%)	100 (30%) NS 80 (80%)	MERS vs non-MERS SARI	66%	31%	NA
(*19*)	59 (median)	NR	17 (55%) NS 13 (77%)	6 (19%) NS 4 (75%)	NA	NA	NA	70%
(*20*)	45 (median)	NR	9 (31%) NS 7 (78%)	8 (28%) NS 8 (100%)	NA	NA	NA	35%
(*21*)	54 (median)	12 (86%)	6 (43%)	6 (42%) 3 (21%) on dialysis	NA	NA	NA	64% 90 d, 43% 28 d
(*22*)	54 (median)	36 (71%)	17 (33%) NS 8 (47%)	14 (28%) had ESRD NS 8 (57%)	NA	NA	NA	37%
(*23*)	48 (mean)	NR	NR	NR	NA	NA	NA	25%
(*24*)	56 (median macrolides); 58 (median no macrolides)	106 (78%) vs. 175 (82%)	72 (53%) vs. 98 (46%)	41 (30%) vs. 68 (32%)	Macrolides	60%	70%	66%
(*25*)	60 (median NIV); 58 (median IMV)	88 (84%) vs. 164 (83%)	62 (59%) vs. 95 (48%)	31 (30%) vs. 68 (35%)	NIV	69%	76%	73%
(*26*)	58 (median RBV/IFN); 58 (no RBV/IFN)	121 (84%) vs. 160 (78%)	84 (58%) vs. 86 (42%)	53 (37%) vs. 56 (27%)	RBVIFN	74%	62%	66%
Case series without evaluation of treatment								
(*27*)	58 (mean)	5 (100%)	4 (80%)	5 (100%)	NA	NA	NA	100%
(*28*)	59 (median)	NR	8 (67%)	5 (42%)	NA	NA	NA	58% at 90 d, 42% at 28 d
(*29*)	57 vs 52 (median)	NR	52 (49%)	21 (20%)	NA	39%	54%	51%
(*30*)	59 (mean)	3 (50%)	0 (0%)	0 (0%)	NA	NA	NA	60%
(*31*)	58 (mean)	NR	NR	NR	NA	NA	NA	63% (0% in included patients)

*CKD, chronic kidney disease; ESRD, end-stage renal disease; HCW, health-care workers; IFN, interferon; IMV, invasive mechanical ventilation; MERS, Middle East respiratory syndrome; NIV, noninvasive ventilation; NR, not reported; NS, nonsurvivors; NA, not applicable; RBV, ribavirin; SARI, severe acute respiratory infection.

### Specific Treatments

These 20 studies collectively examined 11 different pharmaceutical treatments for MERS: 6 antiviral drugs, 2 antibody-mediated therapies, 2 immunomodulatory medications, and 1 antibiotic with possible immunomodulatory effects (*33*) (Appendix Table 1). The studies also examined 2 specific methods of supportive care: NIV and ECMO (Appendix Table 2). Narrative description of studies based on intervention and effect on primary outcome is shown in Table 3. No studies included data on secondary outcomes by treatment provided, and so only mortality rate is described in this report. RoB is shown for all studies in Table 4. Unless otherwise specified, all comparisons described are between patients who received a treatment versus patients who did not (controls).

**Table 3:** Narrative summary of treatments for MERS in humans, based on a systematic review of evidence for MERS treatment with pharmacologic and supportive therapies*

Reference	Patients treated, no.	Study type	Specifics of intervention or analysis	RoB	Outcome†	Certainty of evidence
Ribavirin						
(*22*)	19	Retrospective cohort study	Multivariate logistic regression	Serious	aOR 0.66, 95% CI 0.04–12.36, p = 0.78	Very low evidence; no benefit
(*20*)	10	Retrospective cohort study	Unadjusted	Critical	3/10 (30%) vs. 7/19 (37%), p = 1.0	
Interferons: IFN-α2a, IFN-α2b, INF-β1a						
(*12*)	13 (IFN-α2a); 11 (IFN-β1a)	Nonrandomized single-arm intervention	IFN-α2a vs IFN-β1a; all co-treated with RBV; unadjusted	Serious	aOR (IFN-α) 0.16, 95% CI 0.02–1.38, p = 0.09; aOR (IFN-β) 0.28, 95% CI 0.03–2.33, p = 0.24	Very low evidence; no benefit of IFN-α2a or IFN-β1a
(*22*)	8 (IFN-α); 23 (IFN-β)	Retrospective cohort	Multivariate logistic regression	Serious	aOR 0.47, 95% CI 0.02–10.4, p = 0.63 (IFN-α); aOR 0.68, 95% CI 0.04–10.3, p = 0.78 (IFN-β)	
(*20*)	19	Retrospective cohort	Unadjusted	Critical	6/19 (32%) vs. 4/10 (40%), p = 0.70	
Ribavirin and IFN						
(*26*)	144	Retrospective cohort	Cox-proportional hazards model Marginal structural model	Moderate	aHR 1.52, 95% CI 1.13–2.06, p = 0.006; aOR 1.03, 95% CI 0.73–1.44, p = 0.87	Low evidence no benefit; very low evidence harm
(*14*)	20	Nonrandomized single-arm intervention	Unadjusted	Serious	14d: 6/20 (30%) vs. 17/24 (71%), p = 0.04; 28d: 14/20 (70%) vs. 20/24 (83%), p = 0.05	
Corticosteroids						
(*17*)	151	Retrospective cohort	Marginal structural model	Moderate	aOR (mortality rate) 0.75; 95% CI 0.52–1.07, p = 0.12; aHR (RNA clearance) 0.35; 95% CI 0.17–0.72, p = 0.005	Low evidence no benefit; very low evidence harm
(*22*)	5	Retrospective cohort	Multivariate logistic regression	Serious	aOR 2.92, 95% CI 0.1–63.6, p = 0.5	
(*15*)	1	Prospective cohort	Unadjusted	Critical	0/24 (0%) vs. 1/6 (17%), p = 0.2	
(*23*)	NI	Retrospective cohort	Multivariate logistic regression Paper lacking data	Critical	aOR 3.84, 95% CI 1.95–7.57, p<0.0001	
Macrolide therapy						
(*24*)	136	Retrospective cohort	Multivariate logistic regression Cox-proportional hazards model	Moderate	aOR (mortality rate) 0.84, 95% CI 0.47–1.51, p = 0.56; aHR (RNA clearance) 0.88, 95% CI 0.47–1.64, p = 0.68	Low evidence no benefit
Mycophenolate mofetil						
(*22*)	8	Retrospective cohort	Unadjusted	Serious	8/8 (100%) vs. 0/19 (0%), p = 0.02	Very low evidence of benefit
IVIG						
(*18*)	23	Retrospective cohort	Unadjusted	Moderate	7/113 (6%) vs. 16/217 (7%), p = 0.7	Very low evidence of harm
(*15*)	3	Prospective cohort	Unadjusted	Critical	3/6 (50%) vs. 0/2 (0%), p = 0.005	
Convalescent plasma						
(*16*)	2	Retrospective cohort	Unadjusted	Critical	1/24 (4%) vs. 1/6 (17%), p = 0.37	Very low evidence no benefit
Extracorporeal membrane oxygenation						
(*13*)	17	Nonrandomized single-arm intervention	Unadjusted	Moderate	11/17 (65%) vs. 18/18 (100%), p = 0.02	Low evidence of benefit
(*15*)	2	Retrospective cohort	Unadjusted	Critical	1/24 (4%) vs. 1/6 (17%), p = 0.4	
Noninvasive ventilation						
(*25*)	105	Retrospective cohort	Multivariate logistic regression	Moderate	aOR 0.61, 95% CI 0.23–1.6, p = 0.27	Low evidence no benefit

*Narrative description was decided through consensus among authors based on RoB, type of study, and numbers of patients treated. aHR, adjusted hazard ratio; aOR; adjusted odds ratio; IFN, interferon; IVIg, intravenous immunoglobulin; NI, no information; RoB, risk of bias. †Percentages in parentheses indicate mortality rates.

**Table 4:** Summary of RoB for all single-arm intervention and cohort studies calculated using the ROBBINS-I tool in a systematic review of evidence for MERS treatment with pharmacologic and supportive therapies*

Reference	Reason for RoB determination							
	Confounding	Selection of participants	Classification of interventions	Deviations from intended interventions	Missing outcome data	Outcome measurements	Selection of results reported	Overall RoB
Nonrandomized, single-arm intervention study with historical comparisons								
(*12*)	Serious	Low	Low	Low	Moderate	Low	Moderate	Serious
(*13*)	Moderate	Moderate	Moderate	Moderate	Low	Low	Moderate	Moderate
(*14*)	Serious	Low	Low	Moderate	Low	Low	Moderate	Serious
Prospective cohort study								
(*15*)	Critical	Moderate	Low	Moderate	Low	Low	Moderate	Critical
(*16*)	Serious	Low	Moderate	Moderate	Low	Low	Moderate	Serious
Retrospective cohort study								
(*17*)	Moderate	Low	Low	Moderate	Low	Low	Moderate	Moderate
(*18*)	Moderate	Low	Low	Moderate	Low	Low	Moderate	Moderate
(*19*)	Critical	Serious	Moderate	Moderate	Low	Low	Moderate	Critical
(*20*)	Critical	Moderate	Moderate	Moderate	Low	Low	Moderate	Critical
(*21*)	Critical	Moderate	Moderate	Moderate	Low	Low	Moderate	Critical
(*22*)	Serious	Moderate	Moderate	Moderate	Low	Low	Moderate	Serious
(*23*)	Critical	Serious	Serious	NI	NI	Low	Serious	Critical
(*24*)	Moderate	Low	Low	Low	Low	Low	Moderate	Moderate
(*25*)	Moderate	Moderate	Low	Moderate	Low	Low	Moderate	Moderate
(*26*)	Moderate	Low	Low	Moderate	Low	Low	Moderate	Moderate
Case series without evaluation of treatments								
(*27*)	NA	NA	NA	NA	NA	NA	NA	NA
(*28*)	NA	NA	NA	NA	NA	NA	NA	NA
(*29*)	NA	NA	NA	NA	NA	NA	NA	NA
(*30*)	NA	NA	NA	NA	NA	NA	NA	NA
(*31*)	NA	NA	NA	NA	NA	NA	NA	NA

*NA, not applicable; NI, no information; RoB, risk of bias.

### Specific Antiviral Drugs

Four types of antiviral drugs were used for treatment of MERS in the 20 included studies: lopinavir/ritonavir, oseltamivir, ribavirin, and interferons (α2a, α2b, and β1a). Lopinavir/ritonavir was only used in a single study (*15*), and all patients were treated with the combination, so the effect on the mortality rate could not be elucidated. Oseltamivir was used in most the studies, probably as empiric treatment for influenza. Outcome data were only reported from a single study (*18*) in which authors reported no difference in the crude 90-day mortality rate for patients treated with oseltamivir (112/177 [63%] vs. 105/213 [49%]; p = 0.31).

#### Ribavirin

Outcome data for ribavirin were available in 7 studies (*14*,*18*–*22*,*26*); 3 smaller studies (*18*,*19*,*21*) overlapped with other patient datasets, so we abstracted outcomes from a subsequent larger study (*26*). The effect of ribavirin combined with interferon (IFN) on the mortality rate, as studied by Arabi et al. (*26*) and Omrani et al. (*14*), is described separately. In a retrospective cohort study, Al Ghamdi et al. (*22*) found no association of ribavirin treatment with the crude mortality rate (6/19 [32%] vs. 13/32 [41%]; p = 0.56). Multivariate logistic regression indicated no association of ribavirin treatment with the mortality rate (adjusted odds ratio [aOR] 0.66, 95% CI 0.04–12.36; p = 0.78). This study was at serious RoB because of residual confounding and small sample size.

Sherbini et al. (*20*) found no difference in the mortality rate for patients treated with ribavirin (3/10 [30%] vs. 7/19 [37%]; p = 1.0). This study was at critical RoB because of unmeasured and uncontrolled confounding. Another study (*15*) used ribavirin in all patients, precluding determination of a treatment effect, whereas a final study (*23*) found that ribavirin was not associated with the mortality rate in the patient cohort studied, but no additional data were provided.

#### IFN

Outcomes data for treatment with IFN were available for 8 studies (*12*,*14*,*18*–*22*,*26*); 3 smaller studies (*18*,*19*,*21*) overlapped with other datasets, so we abstracted outcomes from a subsequent larger study (*26*). In a retrospective cohort study, Arabi et al. (*26*) studied the effect of ribavirin and IFN on the 90-day mortality rate in patients with MERS; 144/349 patients (41%) were treated with ribavirin/IFN (58% IFN-α2a, 17% IFN-α2b, and 27% IFN-β1a). No information was available on the mortality rate for each type of IFN. The crude mortality rate was higher in patients treated with ribavirin/IFN (106/144 [74%] vs. 126/205 [62%]; p = 0.02). However, after adjustment for time-varying confounders, ribavirin/IFN treatment was not associated with the 90-day mortality rate (aOR 1.03, 95% CI 0.73–1.44; p = 0.87) or clearance of MERS-CoV RNA (adjusted hazard ratio [aHR] 0.65, 95% CI 0.3–1.44; p = 0.29). This study was at moderate overall RoB.

In a nonrandomized single-arm intervention study, Shalhoub et al. (*12*) compared IFN-α2a with IFN-β1a, where all patients were co-treated with ribavirin. No difference was observed in the unadjusted mortality rate (11/13 [85%] vs. 7/11 [64%]; p = 0.24), or adjusted mortality rate using multivariable models (aOR [IFN-α] 0.16, 95% CI 0.02–1.38; p = 0.09; aOR [IFN-β] 0.28, 95% CI 0.03–2.33, p = 0.24). This study is at serious RoB because of uncontrolled confounding and selection bias, as well as exclusion of patients crossing over from 1 treatment to another. 

In a nonrandomized single-arm intervention study, Omrani et al. (*14*) compared ribavirin/IFN-α2a with supportive care, finding a significantly lower crude 14-day mortality rate for ribavirin/IFN-α2a (6/20 [30%] vs. 17/24 [71%]; p = 0.04) but not a significantly lower crude 28-day mortality rate (14/20 [70%] vs. 20/24 [83%]; p = 0.054). The study was at serious RoB because of selection of patients and unmeasured confounding.

Al Ghamdi et al. (*22*) performed a retrospective cohort study in which 8 patients were treated with IFN-α and 23 patients with IFN-β. They found no association between IFN-α and the crude mortality rate (2/8 [25%] vs. 17/43 [40%]; p = 0.69), but they observed an increase in the crude mortality rate in patients treated with IFN-β (5/23 [22%] vs. 14/28 [50%]; p = 0.05). However, multivariable logistic regression adjusting for severity of illness found no association between IFN-α or IFN-β and the mortality rate (aOR [IFN-α] 0.47, 95% CI 0.02–10.4; p = 0.63; aOR [IFNβ] 0.68, 95% CI 0.04–10.3; p = 0.78). This study had a serious RoB because of the high likelihood of residual confounding and small sample size.

In a retrospective cohort study, Sherbini et al. (*20*) found no difference in the mortality rate among patients treated with IFN (6/19 [32%] vs. 4/10 [40%]; p = 0.7). This study was at critical risk for bias because of unmeasured and uncontrolled confounding. Another study (*15*) used IFN-α2a in all patients in the cohort, whereas a final study (*23*) stated that IFN was not associated with the mortality rate, but no additional data were provided.

### Immunomodulatory Medications

#### Corticosteroids

Eight studies reported outcomes for patients treated with corticosteroids of varying amounts and types (*15*,*17*–*23*); 3 studies (*18*,*19*,*21*) were subsets of another larger study (*17*). In a retrospective cohort study, Arabi et al. (*17*) found that patients who received corticosteroids had a higher crude 90-day mortality rate (112/151 [74%] vs. 91/158 [58%]; p = 0.002). However, by using marginal structural modeling to account for time-varying confounders, they found that corticosteroid therapy was not associated with the 90-day mortality rate (aOR 0.75, 95% CI 0.52–1.07; p = 0.12) and was associated with longer time to MERS-CoV RNA clearance (aHR 0.35, 95% CI 0.17–0.72; p = 0.005). This study was at moderate RoB because it used modeling techniques to control for known confounders.

In a retrospective cohort study, Al Ghamdi et al. (*22*) reported no association between the mortality rate and treatment with hydrocortisone (2/5 [40%] vs. 17/46 [37%]; p = 0.35). They also found no association when adjusting for severity of illness (aOR 2.92, 95% CI 0.1–63.6; p = 0.5). This study was at serious RoB because of a high likelihood of residual confounding. In a prospective cohort study, Hong et al. (*15*) reported on 30 patients with MERS in South Korea. Only 1 patient was treated with corticosteroids, and no association with the mortality rate was observed (1/1 [100%] vs. 5/29 [17%]; p = 0.2). This study was at critical RoB because of unmeasured confounders and bias in participant selection.

In a retrospective cohort study, Alfaraj et al. (*23*) reported that corticosteroids were associated with an increased mortality rate (aOR 3.84, 95% CI 1.95–7.57; p<0.0001), but no further details were provided. Lack of information prevented scoring for all domains of bias, but the paper was judged to be at critical RoB overall because of its uncontrolled design and unmeasured confounding. In a study by Sherbini et al. (*20*), no outcome data could be assessed because all patients were treated with corticosteroids.

#### Macrolides

Mortality rates for patients treated with macrolide therapy were described in 2 studies (*20*,*24*), but 1 study (*20*) was a subset of the other (*24*). In a retrospective cohort study, Arabi et al. (*24*) examined the association of macrolide therapy with the 90-day mortality rate by using multivariable logistic regression and on MERS-CoV RNA plasma clearance by using a Cox proportional hazards model. Macrolide therapy was not independently associated with the mortality rate (aOR 0.84, 95% CI 0.47–1.51; p = 0.56) or RNA clearance (aHR 0.88, 95% CI 0.47–1.64; p = 0.68). This study was at moderate overall RoB given the use of regression models to attempt to account for confounding.

#### Mycophenolate Mofetil

Mycophenolate mofetil was only used in a single study by Al Ghamdi et al. (*22*) and was associated with a decrease in the crude mortality rate (0/8 [0%] vs. 19/43 [44%]; p = 0.02). Mycophenolate mofetil could not be evaluated in a multivariable model because all patients survived. This study was at serious RoB because of the high likelihood of residual confounding.

### Antibody-Mediated Pharmaceuticals

#### Intravenous Immunoglobulin and Convalescent Plasma

Mortality rates for patients treated with intravenous immunoglobulin (IVIg) were reported in 2 studies (*15*,*18*). One retrospective cohort study (*18*) at moderate RoB reported no association between IVIg and the mortality rate (16/23 [70%] vs. 201/307 [65%]; p = 0.69). Another study (*15*) at critical RoB reported a significant increase in the mortality rate in patients treated with IVIg (3/3 [100%] vs. 3/27 [11%]; p = 0.005). A single study (*15*) at critical RoB reported no association between treatment with convalescent plasma and the mortality rate (1/2 patients [50%] vs. 5/28 [18%]; p = 0.37).

### Supportive Care

#### ECMO

Six studies reported outcome data for treatment of MERS with ECMO (*13*,*15*,*16*,*18*,*19*,*22*). One study (*13*) looking specifically at ECMO provided more detailed information and captured all ECMO-treated patients included in 4 other studies (*16*,*18*,*19*,*22*). In a nonrandomized, single-arm intervention study, Alshahrani et al. (*13*) reported a lower mortality rate among patients treated with ECMO versus supportive care (11/17 [65%] vs. 18/18 [100%]; p = 0.02). The study attempted to control for bias by identifying patients in the pre-ECMO period who would have been eligible for ECMO if available. The study was still at moderate overall RoB because of unmeasured and unknown confounding. Hong et al. (*15*) found no association between ECMO and the mortality rate at any time point (1/2 [50%] vs. 5/28 [4%]; p = 0.4); however, this study was at critical RoB.

#### NIV

Three studies reported mortality rates for MERS patients treated with NIV (*18*,*19*,*25*), but 2 studies (*18*,*19*) were subsets of another study (*25*). In a retrospective cohort study, Alraddadi et al. (*25*) reviewed the cases of patients who were initially managed with NIV (105/302 [35%]) compared with those managed with invasive ventilation alone. Most (92%) of the NIV group required invasive mechanical ventilation. NIV was not independently associated with the 90-day mortality rate (aOR 0.61, 95% CI 0.23–1.6; p = 0.27). This study was at moderate overall RoB because it used propensity scores to adjust for known confounders.

### Case Series

No meaningful outcome data based on specific treatments or supportive care could be derived from any of the case series. This lack of data was attributable to inadequate reported information or because all patients in the case series were treated with identical therapies.

### Overall Certainty of Evidence

In terms of assessing MERS patient mortality rates, the certainty of evidence is low or very low because all 20 studies had at least moderate RoB and because of the imprecision of estimates of treatment effect. We did not downgrade for inconsistency because meta-analyses were not possible and statistical heterogeneity could not be assessed. Studies generally had appropriate inclusion criteria for MERS patients and therefore provided direct evidence. We found no evidence that might suggest publication bias.

## Discussion

In this systematic review we identified 3 nonrandomized single-arm intervention studies, 12 cohort studies, and 5 case series evaluating specific treatments and supportive care for MERS patients. Most studies were at serious or critical RoB because of confounding and selection bias.

Low-quality evidence suggests no benefit from corticosteroids or combination of ribavirin with any type of IFN but also very low evidence of harm. Low-quality evidence from a single study suggests no benefit from macrolide therapy. Low-quality evidence indicated a benefit from ECMO for severe MERS cases from a single study. Low-quality evidence suggests no benefit from NIV. All other treatments assessed had very low-quality of evidence. On the basis of this review, no specific pharmacologic therapies have sufficient evidence of effectiveness to warrant a treatment recommendation, although ECMO might be considered for severe MERS.

Our study has several strengths, including a broad review of the published literature, assessment of RoB according to the Cochrane framework, and duplicate independent data extraction. Although ours is not the first systematic review of treatment for MERS, we report on a large number of patients (*34*,*35*). We estimate the number of unique cases to be ≈678–865. Our study also specifically evaluated both pharmacologic treatments and supportive care for MERS.

Our study has limitations. We are limited in any inferences we can draw from these reviewed studies because of substantial RoB and low-quality of evidence in most publications. Few studies evaluating a single intervention, the substantial heterogeneity in study populations and design, and overlap in patient populations precluded meta-analyses. Assessing the effect of pharmaceutical interventions is challenging because of the substantial heterogeneity in timing and dose of treatments administered. Also, many studies had no contemporaneous similar comparator group, and most were retrospective in nature. Overall, the quality of evidence bearing on any individual treatment we reviewed was very low to low, owing to RoB and the imprecision of included studies. Ongoing research might provide additional rigorous data on specific treatments (e.g., the MIRACLE trial, an RCT of lopinavir/ritonavir and IFN-β1b [https://clinicaltrials.gov/ct2/show/NCT02845843]).

Several treatment options in earlier phases of clinical study were not within the scope of this systematic review and did not meet inclusion criteria for our study but are worth highlighting as potential future directions of research. Many of these studies have been included in prior systematic reviews of preclinical studies (*35*). One example is SAB-301, a polyclonal antibody directed at the MERS-CoV spike protein that is derived from transchromosomic cows. A phase 1 trial published in 2018 demonstrated the safety and tolerability of this treatment (*36*). Another example is a phase 1 trial from 2019 that demonstrated the safety and tolerability of the GLS-5300 MERS coronavirus vaccine in humans (*37*). Other potential treatment options still in early phases of development have been summarized elsewhere (*38*,*39*). The high mortality rate, lack of proven effective treatments, ongoing potential for human-to-human transmission, and the emergence of novel coronaviruses (*40*) underscore the importance of developing research capacity in regions prone to MERS outbreaks as well as the capacity to perform collaborative clinical trials to improve the treatment evidence base.

In this systematic review of potential therapies for MERS, we found existing studies to be at moderate to critical RoB. Low-quality evidence (based on a single study) indicates a benefit from ECMO in severe MERS cases. Low-quality evidence also exists showing no benefit of corticosteroids, NIV, macrolides, or combination of ribavirin with any type of IFN. Collaborative clinical trials evaluating potential therapies are urgently needed to guide treatment decisions (*41**,**42*).

Appendix Additional information about pharmacologic treatments and supportive care for Middle East respiratory syndrome.
