# Supplementary material for: Pharmacologic Treatments and Supportive Care for Middle East Respiratory Syndrome
Source: Emerg Infect Dis. 2020 Jun;26(6):1102–12. doi: 10.3201/eid2606.200037 (PMC7258456; doi:10.3201/eid2606.200037)
Supplement: Appendix — Additional information about pharmacologic treatments and supportive care for Middle East respiratory syndrome. [file 20-0037-Techapp-s1.pdf]

Article DOI: https://doi.org/10.3201/eid2606.200037

Pharmacologic Treatments and Supportive Care for Middle East Respiratory Syndrome

Appendix

Appendix Table 1. Summary of specific treatments used by clinicians for the treatment of MERS-CoV in humans in each of the studies. Number of patients who did not survive (NS) and received each therapy reported where available*

	Patients	Specific Treatments										
Reference	(n)	Ostmvr	Ribavirin	IFN-α2a	IFN-α2b	IFN-β1a	Steroids	CvP	MMF	Macrolides	IVIG	Lopi/r
Nonrandomized, Single-Arm Intervention Study with Historical Comparisons												
(*1*)	32	0	24 (75%) NS 19 (79%)	13 (41%) NS 11 (85%)	0	11 (34%) NS 7 (64%)	0	0	0	0	0	0
(*2*)	35	0	14 (83% ECMO) 4 (29% no ECMO)	11 (65% ECMO) 4 (36% no ECMO)	0	0	9 (53% ECMO) 13 (72% no ECMO)	0	0	0	0	0
(*3*)	44	33 (73%)	20 (46%)	20 (46%)	0	0	23 (52%)	0	0	0	0	0
Prospective Cohort Study												
(*4*)	30	0	30 (100%) NS 6 (20%)	30 (100%) NS 6 (20%)	0	0	1 (3%) NS 1 (100%)	2 (7%) NS 1 (100%)	0	0	3 (10%) NS 3 (100%)	0
(*5*)	8	8 (100%) NS 5 (63%)	8 (100%) NS 5 (63%)	8 (100%) NS 5 (63%)	0	0	8 (100%) NS 5 (63%)	0	0	0	0	0
Retrospective Cohort Study												
(*6*)	309	169 (55%)	127 (41%)	119 (39%)	0	0	151 (49%) NS 114 (76%)	0	0	0	0	0
(*7*)	330	177 (54%) NS 112 (63%)	133 (40%) NS 96 (72%)	124 (38%) NS 104 (84%)	0	0	177 (54%) NS 133 (75%)	0	0	0	23 (7%) NS 16 (70%)	0
(*8*)	31	20 (65%) NS 15 (75%)	13 (42%) NS 9 (69%)	13 (42%) NS 9 (69%)	0	0	8 (26%) NS 6 (75%)	0	0	0	0	0
(*9*)	29	0	10 (35%) NS 3 (30%)	19 (66%) NS 6 (32%)	0	0	29 (100%) NS 10 (35%)	0	0	19 (66%) NS 6 (32%)	0	0
(*10*)	14	7 (50%)	11 (79%) NS 6 (55%)	11 (79%) NS 6 (55%)	0	0	14 (100%) NS 9 (64%)	0	0	0	0	0
(*11*)	51	0	19 (38%) NS 6 (32%)	8 (16%) NS 2 (25%)	0	23 (45%) NS 5 (22%)	5 (10%) NS 3 (60%)	0	8 (16%) NS 0	0	0	0
(*12*)	314	NR	NR	NR	NR	NR	NR	0	0	NR	0	0

Reference	Patients (n)	Specific Treatments										
		Ostmvr	Ribavirin	IFN-α2a	IFN-α2b	IFN-β1a	Steroids	CvP	MMF	Macrolides	IVIG	Lopi/r
(*13*)	349	196 (56%)	NR	NR	NR	NR	178 (51%)	0	0	136 (39%)	24 (7%)	NR
(*14*)	302	NR	NR	NR	NR	NR	NR	NR	NR	NR	NR	NR
(*15*)	349	196 (56%)	122 (35%)	73 (58%)	22 (18%)	40 (11%)	178 (51%)	NR	NR	NR	NR	NR
Case Series without Evaluation of Treatment												
(*16*)	5	5 (100%)<br>NS 5 (100%)	5 (100%)<br>NS 5 (100%)	0	5 (100%)<br>NS 5 (100%)	0	5 (100%)<br>NS 5 (100%)	0	0	0	0	0
(*17*)	12	7 (58%)	0	0	0	0	10 (83%)	0	0	0	1 (8%)	0
(*18*)	107	13 (12%)	61 (57%)	47 (43%)	54 (51%)	48 (45%)	24 (22%)	14 (13%)	22 (21%)	59 (55%)	33 (31%)	41 (38%)
(*19*)	6	0	6 (100%)<br>NS 3 (50%)	6 (100%)<br>NS 3 (50%)	0	0	1 (17%)<br>NS 0	0	0	0	0	0
(*20*)	63 (8 included)	0	0	0	0	0	0	0	0	0	0	0

*NS, non-survivors; Ostmvr, Oseltamavir; MMF, Mycophenolate mofetil; IFN, interferon; IVIG, intravenous immunoglobulins; CvP, Convalescent Plasma; Lopi/r, Lopinavir/ritonavir; NR, not reported.

Appendix Table 2. Summary of supportive care used by clinicians for the treatment of MERS-CoV in humans in each of the studies. Where available table shows unadjusted number of patients receiving therapy in each arm of the study with p values reported by authors. Number of patients who did not survive (NS) and received each therapy reported where available*

Reference	Patients (*n*)	Supportive Care						
		NIV	Mechanical Ventilation	NMB	Nitric Oxide	ECMO	Vasopressors	RRT
Nonrandomized, Single-Arm Intervention Study with Historical Comparisons								
(*1*)	32	NR	16 (50%)	NR	NR	NR	NR	8 (25%)
(*2*)	35	0	35 (100%)	NR	NR	17 (49%)	NR	NR
(*3*)	44	3 (7%)	41 (93%)	NR	NR	4 (9%)	32 (73%)	22 (50%)
Prospective Cohort Study								
(*4*)	30	NR	8 (27%)<br>NS 6 (75%)	NR	NR	2 (7%)<br>NS 2 (100%)	NR	2 (7%)<br>NS 1 (50%)
(*5*)	8	5 (63%)	7 (88%)	NR	NR	1 (13%)<br>NS 0	NR	NR
Retrospective Cohort Study								
(*6*)	309	54 (36%) vs 41 (26%) ($p = 0.06$)	141 (93%) vs 121 (77%) ($p < 0.0001$)	74 (49%) vs 46 (29%) ($p = 0.0003$)	29 (19%) vs 10 (6%) ($p = 0.0007$)	10 (7%) vs 8 (5%) ($p = 0.56$)	134 (89%) vs 111 (70%) ($p < 0.0001$)	83 (55%) vs 69 (44%) ($p = 0.05$)
(*7*)	330	100 (30%)<br>NS 63 (63%)	281 (85%)<br>NS 204 (73%)	133 (40%)<br>NS 97 (73%)	43 (13%)<br>NS 34 (79%)	19 (6%)<br>NS 13 (68%)	262 (79%)<br>NS 199 (76%)	161 (49%)<br>NS 131 (81%)
(*8*)	31	14 (45%)<br>NS 8 (57%)	27 (87%)<br>NS 22 (82%)	12 (39%)<br>NS 10 (83%)	NR	1 (3%)<br>NS 1 (100%)	25 (81%)<br>NS 22 (88%)	16 (52%)<br>NS 14 (88%)
(*9*)	29	NR	9 (31%)<br>NS 8 (89%)	NR	NR	NR	NR	NR
(*10*)	14	2 (14%)	14 (100%)	11 (79%)	8 (57%)	0	12 (86%)	8 (57%)

Reference	Patients (*n*)	Supportive Care						
		NIV	Mechanical Ventilation	NMB	Nitric Oxide	ECMO	Vasopressors	RRT
(*11*)	51	NR	NR	NR	NR	10 (19.6%)<br>NS 10 (100%)	NR	NR
(*12*)	314	NR	NR	NR	NR	NR	NR	NR
(*13*)	349	49 (36%) vs. 57 (27%) (p = 0.07)	117 (86%) vs. 180 (85%) (p = 0.7)	59 (43%) vs. 74 (35%) (p = 0.11)	23 (17%) vs. 21 (10%) (p = 0.05)	10 (7%) vs. 12 (6%) (p = 0.52)	107 (78%) vs. 169 (79%) (p = 0.88)	79 (58%) vs. 95 (45%) (p = 0.01)
(*14*)	302	105 (35%)	294 (97%)	51 (49%) vs. 82 (42%) (p = 0.25)	21 (20%) vs. 23 (12%) (p = 0.05)	11 (11%) vs. 11 (6%) (p = 0.12)	88 (84%) vs. 177 (90%) (p = 0.13)	49 (47%) vs. 115 (58%) (p = 0.05)
(*15*)	349	50 (35%) vs. 56 (27%) (p = 0.14)	126 (88%) vs. 171 (83%) (p = 0.29)	46 (32%) vs. 87 (42%) (p = 0.05)	20 (14%) vs. 24 (12%) (p = 0.55)	11 (8%) vs. 11 (5%) (p = 0.39)	111 (77%) vs. 165 (81%) (p = 0.44)	74 (51%) vs. 100 (49%) (p = 0.63)
Case Series without Evaluation of Treatment								
(*16*)	5	NR	5 (100%)<br>NS 5 (100%)	NR	NR	NR	NR	5 (100%)<br>NS 5 (100%)
(*17*)	12	5 (42%)	12 (100%)	4 (33%)	6 (50%)	0	11 (92%)	7 (58%)
(*18*)	107	NR	56 (52%)	NR	NR	46 (43%)	33 (31%)	31 (29%)
(*19*)	6	1 (17%)<br>NS 0	3 (50%)<br>NS 3 (100%)	NR	NR	NR	3 (50%)<br>NS 3 (100%)	3 (50%)<br>NS 3 (100%)
(*20*)	63 (8 HCWs)	12 (19%)<br>HCWs 2 (25%)	51 (81%)<br>HCWs 8 (100%)	HCWs 7 (88%)	HCWs 2 (25%)	HCWs 1 (13%)	37 (59%)<br>HCWs 7 (88%)	37 (59%)<br>HCWs 4 (50%)

*NS, non-survivors; NIV, non-invasive ventilation; ECMO, extra-corporeal membrane oxygenation; NMB, neuromuscular blockade; RRT, renal replacement therapy; NR, Not reported

Search Terms (Via MEDLINE)
Database: Ovid MEDLINE: Epub Ahead of Print, In-Process & Other Non-Indexed Citations, Ovid MEDLINE® Daily and Ovid MEDLINE® <1946-Present>
Search Strategy:
----
1 exp CORONAVIRUS INFECTIONS/ or exp CORONAVIRUS/ or exp MIDDLE EAST RESPIRATORY SYNDROME CORONAVIRUS/ (13600)
2 middle east respiratory syndrome.tw. (1317)
3 coronavirus.tw. (8584)
4 MERS.tw. (3344)
5 MERS-CoV.mp. (1135)
6 1 or 2 or 3 or 4 or 5 (18103)
7 treatment.mp. or exp Therapeutics/ (7102731)
8 interferon.mp. or exp Interferons/ (185605)
9 ribavirin.mp. or exp RIBAVIRIN/ (15864)
10 lopinavir.mp. or exp LOPINAVIR/ (2572)
11 glucocorticoids.mp. or exp GLUCOCORTICOIDS/ (194948)
12 exp Mycophenolic Acid/ or mmf.mp. (9269)
13 convalescent plasma.mp. (116)
14 exp Antibodies, Neutralizing/ or exp Antibodies, Monoclonal/ or neutralizing monoclonal antibody.mp. (221498)
15 IVIG.mp. or exp Immunoglobulins, Intravenous/ (14075)
16 exp Spike Glycoprotein, Coronavirus/ (1147)
17 6 or 16 (18114)
18 supportive care.mp. (13380)
19 mechanical ventilation.mp. or exp Respiration, Artificial/ (88846)
20 exp VACCINES/ or vaccine.mp. (268922)
21 7 or 8 or 9 or 10 or 11 or 12 or 13 or 14 or 15 or 18 or 19 or 20 (7549505)
22 17 and 21 (5138)
23 Humans/ (17200300)
24 22 and 23 (2461)

Appendix Figure. Search strategy used for systematic review as shown in MEDLINE search. Search was repeated throughout 5 databases (MEDLINE, PubMed, Embase, Cochrane, and CINHAHL).

References

1. Shalhoub S, Farahat F, Al-Jiffri A, Simhairi R, Shamma O, Siddiqi N, et al. IFN-α2a or IFN-β1a in combination with ribavirin to treat Middle East respiratory syndrome coronavirus pneumonia: a retrospective study. J Antimicrob Chemother. 2015;70:2129–32. PubMed https://doi.org/10.1093/jac/dkv085

2. Alshahrani MS, Sindi A, Alshamsi F, Al-Omari A, El Tahan M, Alahmadi B, et al. Extracorporeal membrane oxygenation for severe Middle East respiratory syndrome coronavirus. Ann Intensive Care. 2018;8:3. PubMed https://doi.org/10.1186/s13613-017-0350-x

3. Omrani AS, Saad MM, Baig K, Bahloul A, Abdul-Matin M, Alaidaroos AY, et al. Ribavirin and interferon alfa-2a for severe Middle East respiratory syndrome coronavirus infection: a retrospective cohort study. Lancet Infect Dis. 2014;14:1090–5. PubMed https://doi.org/10.1016/S1473-3099(14)70920-X

4. Hong KH, Choi JP, Hong SH, Lee J, Kwon JS, Kim SM, et al. Predictors of mortality in Middle East respiratory syndrome (MERS). Thorax. 2018;73:286–9. PubMed https://doi.org/10.1136/thoraxjnl-2016-209313

5. Al-Hameed F, Wahla AS, Siddiqui S, Ghabashi A, Al-Shomrani M, Al-Thaqafi A, et al. Characteristics and outcomes of Middle East respiratory syndrome coronavirus patients admitted to an intensive care unit in Jeddah, Saudi Arabia. J Intensive Care Med. 2016;31:344–8. PubMed https://doi.org/10.1177/0885066615579858

6. Arabi YM, Mandourah Y, Al-Hameed F, Sindi AA, Almekhlafi GA, Hussein MA, et al.; Saudi Critical Care Trial Group. Corticosteroid therapy for critically ill patients with Middle East respiratory syndrome. Am J Respir Crit Care Med. 2018;197:757–67. PubMed https://doi.org/10.1164/rccm.201706-1172OC

7. Arabi YM, Al-Omari A, Mandourah Y, Al-Hameed F, Sindi AA, Alraddadi B, et al.; Saudi Critical Care Trial Group. Critically ill patients with the Middle East respiratory syndrome: a multicenter retrospective cohort study. Crit Care Med. 2017;45:1683–95. PubMed https://doi.org/10.1097/CCM.0000000000002621

8. Almekhlafi GA, Albarrak MM, Mandourah Y, Hassan S, Alwan A, Abudayah A, et al. Presentation and outcome of Middle East respiratory syndrome in Saudi intensive care unit patients. Crit Care. 2016;20:123. PubMed https://doi.org/10.1186/s13054-016-1303-8

9. Sherbini N, Iskandrani A, Kharaba A, Khalid G, Abduljawad M, Al-Jahdali H. Middle East respiratory syndrome coronavirus in Al-Madinah City, Saudi Arabia: demographic, clinical and survival data. J Epidemiol Glob Health. 2017;7:29–36. PubMed https://doi.org/10.1016/j.jegh.2016.05.002

10. Khalid I, Alraddadi BM, Dairi Y, Khalid TJ, Kadri M, Alshukairi AN, et al. Acute management and long-term survival among subjects with Middle East respiratory syndrome coronavirus pneumonia and ARDS. Respir Care. 2016;61:340–8. PubMed https://doi.org/10.4187/respcare.04325

11. Al Ghamdi M, Alghamdi KM, Ghandoora Y, Alzahrani A, Salah F, Alsulami A, et al. Treatment outcomes for patients with Middle Eastern respiratory syndrome coronavirus (MERS CoV) infection at a coronavirus referral center in the Kingdom of Saudi Arabia. BMC Infect Dis. 2016;16:174. PubMed https://doi.org/10.1186/s12879-016-1492-4

12. Alfaraj SH, Al-Tawfiq JA, Assiri AY, Alzahrani NA, Alanazi AA, Memish ZA. Clinical predictors of mortality of Middle East respiratory syndrome coronavirus (MERS-CoV) infection: a cohort study. Travel Med Infect Dis. 2019;29:48–50. PubMed https://doi.org/10.1016/j.tmaid.2019.03.004

13. Arabi YM, Deeb AM, Al-Hameed F, Mandourah Y, Almekhlafi GA, Sindi AA, et al.; Saudi Critical Care Trials group. Macrolides in critically ill patients with Middle East respiratory syndrome. Int J Infect Dis. 2019;81:184–90. PubMed https://doi.org/10.1016/j.ijid.2019.01.041

14. Alraddadi BM, Qushmaq I, Al-Hameed FM, Mandourah Y, Almekhlafi GA, Jose J, et al.; Saudi Critical Care Trials Group. Noninvasive ventilation in critically ill patients with the Middle East respiratory syndrome. Influenza Other Respir Viruses. 2019;13:382–90. PubMed https://doi.org/10.1111/irv.12635

15. Arabi YM, Shalhoub S, Mandourah Y, Al-Hameed F, Al-Omari A, Al Qasim E, et al. Ribavirin and interferon therapy for critically ill patients with Middle East respiratory syndrome: a multicenter observational study. Clin Infect Dis. 2019;Jun 25:ciz544. PubMed https://doi.org/10.1093/cid/ciz544

16. Al-Tawfiq JA, Momattin H, Dib J, Memish ZA. Ribavirin and interferon therapy in patients infected with the Middle East respiratory syndrome coronavirus: an observational study. Int J Infect Dis. 2014;20:42–6. PubMed https://doi.org/10.1016/j.ijid.2013.12.003

17. Arabi YM, Arifi AA, Balkhy HH, Najm H, Aldawood AS, Ghabashi A, et al. Clinical course and outcomes of critically ill patients with Middle East respiratory syndrome coronavirus infection. Ann Intern Med. 2014;160:389–97. PubMed https://doi.org/10.7326/M13-2486

18. Alhumaid S, Tobaiqy M, Albagshi M, Alrubaya A, Algharib F, Aldera A, et al. MERS-CoV transmitted from animal-to-human vs MERS-CoV transmitted from human-to-human: comparison of virulence and therapeutic outcomes in a Saudi hospital. Trop J Pharm Res. 2018;17:1155–64. https://doi.org/10.4314/tjpr.v17i6.23

19. Khalid M, Khan B, Al Rabiah F, Alismaili R, Saleemi S, Rehan-Khaliq AM, et al. Middle Eastern respiratory syndrome corona virus (MERS CoV): case reports from a tertiary care hospital in Saudi Arabia. Ann Saudi Med. 2014;34:396–400. PubMed https://doi.org/10.5144/0256-4947.2014.396

20. Al-Dorzi HM, Aldawood AS, Khan R, Baharoon S, Alchin JD, Matroud AA, et al. The critical care response to a hospital outbreak of Middle East respiratory syndrome coronavirus (MERS-CoV) infection: an observational study. Ann Intensive Care. 2016;6:101–12. PubMed https://doi.org/10.1186/s13613-016-0203-z
